# The Association between Red Blood Cell Distribution Width and Mortality in Critically Ill Patients with Acute Kidney Injury

**DOI:** 10.1155/2018/9658216

**Published:** 2018-09-24

**Authors:** Benji Wang, Huaya Lu, Yuqiang Gong, Binyu Ying, Bihuan Cheng

**Affiliations:** ^1^Department of Anesthesiology, Critical Care and Pain Medicine, The Second Affiliated Hospital and Yuying Children's Hospital of Wenzhou Medical University, Wenzhou, Zhejiang 325000, China; ^2^Department of Orthopedics, Ningbo Yinzhou Second Hospital, Ningbo, Zhejiang 315100, China

## Abstract

**Background:**

Several investigators have sought risk factors for mortality in acute kidney injury (AKI). However, no epidemiological studies have investigated the impact of red blood cell distribution width (RDW) on prognosis for critically ill patients with AKI. The aim of this study was to investigate the association of RDW with mortality in these patients.

**Methods:**

We analyzed data from the MIMIC-III. RDW was measured upon ICU admission. The association between RDW and mortality of AKI was determined using a multivariate logistic regression and was expressed as the adjusted odds ratio with associated 95% confidence interval (CI). We also conducted subgroup analyses to determine the consistency of this association.

**Results:**

A total of 14,078 critically ill patients with AKI were eligible for this analysis. In multivariate analysis, adjusted for age and gender and compared with the reference group (RDW 11.1-13.4%) related to hospital mortality, the adjusted ORs (95% CIs) for RDW levels 13.5-14.3%, 14.4-15.6%, and 15.7-21.2% were 1.22 (1.05, 1.43), 1.56 (1.35, 1.81), and 2.66 (2.31, 3.06), respectively. After adjusting for confounding factors, with high RDW linked to an increase in mortality (RDW 15.7-21.2% versus 11.1-13.4%: OR, 1.57; 95% CI, 1.22 to 2.01;* P* trend <0.0001). A similar trend was observed for 30-day mortality.

**Conclusions:**

RDW appeared to be an independent prognostic marker in critically ill patients with AKI and higher RDW was associated with increased risk of mortality in these patients.

## 1. Introduction

Acute kidney injury (AKI) is a common and serious syndrome. It is diagnosed with urine output and serum creatinine criteria and is associated with high morbidity and mortality, especially in critical illness [1, 2]. In addition, survivors often fail to restore kidney function and require long-term dialysis [3, 4], which brings a huge economic burden [5]. Considering the high incidence of AKI and the poor prognosis in critical illness, several investigators have sought risk factors of mortality in AKI [6, 7].

Red blood cell distribution width (RDW) reflects the size variation of circulating red blood cells and is calculated in automated complete blood counts [8]. Previously, the clinical use of RDW was limited to the differential diagnosis of anemia. An increased RDW was considered significantly associated with risk of adverse outcomes in patients with cardiovascular diseases [9, 10], multiple myeloma [11], systemic sclerosis [12], and ischemic stroke [13]. A study demonstrated that RDW could be an additive predictor for mortality in AKI patients undergoing continuous renal replacement therapy (CRRT) [14]. Moreover, a recent study demonstrated that increased RDW was associated with increased risk of AKI and mortality in critically ill patients in the coronary care unit (CCU) [15]. Despite these observations, no epidemiological studies have investigated the impact of RDW on prognosis of critically ill patients with AKI. The aim of this study was to evaluate the association of RDW with mortality in these patients.

## 2. Methods

### 2.1. The Database

Multiparameter Intelligent Monitoring in Intensive Care III version 1.3 (MIMIC-III v1.3) is a public and freely available intensive care unit (ICU) database. It includes more than 50,000 ICU patients (medical, surgical, coronary care, and neonatal) admitted to Beth Israel Deaconess Medical Center (Boston, MA, USA) from 2001 to 2012 [16]. To apply for access to the database, we completed the National Institutes of Health's web-based course and passed the Protecting Human Research Participants exam (no. 6182750). In the present study, we extracted clinical data, including patient demographics and laboratory test results. The database was approved by the Institutional Review Boards of Beth Israel Deaconess Medical Center (Boston, MA) and the Massachusetts Institute of Technology (Cambridge, MA). To safeguard patient privacy, the information of all included patients was hidden.

### 2.2. Population Selection Criteria

The database included a total of 58,976 ICU admissions. Patients with AKI who were over the age of 18 years and had been hospitalized in the ICU at first admission for more than two days were included. Improving Global Outcomes (KDIGO) determined the classification of AKI [17]. Patients were excluded if they met the following criteria: (1) no RDW measured during ICU stay; (2) hematologic disease such as leukemia and myelodysplastic syndrome; and (3) missing > 5% individual data.

### 2.3. Data Extraction

Data extraction was performed by using Structured Query Language (SQL) with PostgreSQL (version 9.6). The following data were extracted: age, gender, ethnicity, BMI, SBP, DBP, respiratory rate, temperature, comorbidities, laboratory parameters, and scoring systems. The following comorbidities were extracted: congestive heart failure (CHF), coronary artery disease (CAD), atrial fibrillation (AF), chronic kidney disease (CKD), chronic liver disease, chronic obstructive pulmonary disease (COPD), stroke, malignancy, respiratory disease (non-COPD), and acute respiratory distress syndrome (ARDS). We extracted the following laboratory parameters: white blood cell (WBC), blood urea nitrogen (BUN), chloride, bilirubin, hematocrit, and hemoglobin. We also calculated sequential organ failure assessment (SOFA) [18] and acute physiology score III (APS III) [19]. Hospital mortality and 30-day all-cause mortality were the clinical outcomes. The baseline characteristic data were extracted within the first 24 h after patient ICU admission and were analyzed in three equal groups.

### 2.4. Statistical Analysis

Baseline characteristics of all patients were stratified by RDW tertiles. Continuous variables were presented as mean ± standard deviation (SD) or medians and compared using the analysis of variance or the Kruskal-Wallis test. Categorical data were summarized as number or percentage.

The associations between RDW and hospital mortality and 30-day all-cause mortality of AKI were determined using a multivariate logistic regression and were expressed as the adjusted odds ratio with associated 95% confidence interval (CI). We used two multivariate models to determine whether there was a significant association of RDW with mortality. We selected these confounders based on their associations with the hospital mortality of RDW or a change in effect estimate of more than 10%. In model I, covariates were adjusted for age and gender. In model II, we adjusted for age, gender, albumin, hemoglobin, liver disease, renal disease, malignancy, bilirubin, WBC, BUN, APS III, SOFA, systemic inflammatory response syndrome (SIRS), renal replacement therapy (RRT), temperature, and Elixhauser [20]. We performed stratification analyses to determine whether the effect of RDW differed across various subgroups classified by age, gender, CHF, AKI stage, AF, COPD, CAD, stroke, respiratory failure, pneumonia, ARDS, liver, vasoactive drug administration RRT, and SIRS. We generated Receiver Operating Characteristic (ROC) curves to measure the sensitivity and specificity of RDW and existing scoring systems (SOFA score or APS III score) and calculated the area under the curve (AUC) to ascertain the quality of RDW as a predictor of mortality.

A two-tailed *P* value <0.05 was considered to be statistically significant. EmpowerStats version 2.17.8 (http://www.empowerstats.com/cn/) and R software version 3.42 were used for all statistical analysis.

## 3. Results

### 3.1. Subject Characteristics

A total of 14,078 critically ill patients with AKI were eligible for this analysis. According to the RDW, there were three equal groups: 4672 patients were in the low-RDW group (11.1-13.7%), 4664 patients were in the mid-RDW group (13.8-15.1%), and 4742 patients were in the high-RDW group (15.2-21.2%). As shown in Table 1, 8086 (57.4%) were men and 9964 (70.8%) were white. Patients in the higher RDW (15.2-21.2%) tended to be elderly, female, and white and were more likely to have a history of CHF, AF, CKD, chronic liver disease, malignancy, respiratory disease (non-COPD), and ARDS. Moreover, these patients had higher BUN and bilirubin and lower hematocrit and hemoglobin. They also had higher SOFA and APS III scores and were more likely to use RRT than were those with low-RDW group (11.1-13.7%).

### 3.2. RDW Levels and Mortality

Higher RDW was associated with increased risk of hospital mortality and 30-day all-cause mortality in critically ill patients with AKI (Table 2). In multivariate analysis, adjusted for age and gender and compared with reference group (RDW 11.1-13.4%), the adjusted ORs (95% CIs) for RDW levels 13.5-14.3%, 14.4-15.6%, and 15.7-21.2% were 1.22 (1.05, 1.43), 1.56 (1.35, 1.81), and 2.66 (2.31, 3.06), respectively. After adjusting for age, gender, albumin, hemoglobin, liver disease, renal disease, malignancy, bilirubin, WBC, BUN, APS III, SOFA, SIRS, RRT, temperature, and Elixhauser, a similar trend was observed: high RDW was associated with an increase in mortality (RDW 15.7-21.2% versus 11.1-13.4%: OR, 1.57; 95% CI, 1.22 to 2.01;* P* trend <0.0001). Meanwhile, a similar trend was observed for 30-day all-cause mortality (Table 2).

### 3.3. Subgroup Analyses

We conducted subgroup analyses to determine the consistency of association between RDW and risk of hospital mortality in critically ill patients with AKI (Table 3). Patients who were female showed an increased risk of hospital mortality with higher RDW (OR 1.08, 95% CI 1.02 to 1.14). Similarly, patients with respiratory failure (OR, 1.09; 95% CI, 1.04 to 1.14), pneumonia (OR, 1.08; 95% CI, 1.02 to 1.14), and treatment with vasoactive agents (OR, 1.09; 95% CI, 1.04 to 1.14) had a significantly higher risk of hospital mortality with higher RDW.

### 3.4. Prediction of Mortality

**Figure 1:**
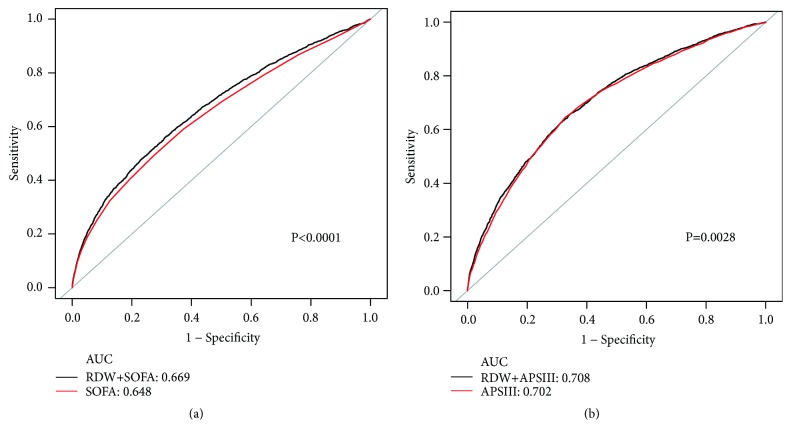
ROC curves for the prediction of mortality in critically ill patients with AKI. (a) The ability of SOFA scores and RDW plus SOFA scores to predict hospital mortality. (b) The ability of APS III scores and RDW plus APS III scores to predict hospital mortality.

ROC curves generated using the indicated variables (SOFA scores, RDW plus SOFA scores and APS III scores, and RDW plus APS III scores) were plotted in Figure 1. The AUC for SOFA scores was 0.648, compared to 0.669 for RDW plus SOFA scores (*P* < 0.0001). Furthermore, the AUCs for the relationships between APS III scores and RDW plus APS III scores were 0.702 and 0.708 (*P* = 0.0028).

## 4. Discussion

We observed that higher RDW was associated with increased risk of hospital mortality and 30-day all-cause mortality in critically ill patients with AKI. Furthermore, after adjusting for age, gender, albumin, hemoglobin, liver disease, renal disease, malignancy, bilirubin, WBC, BUN, APS III, SOFA, SIRS, RRT, temperature, and Elixhauser, the high RDW was associated with an increase in mortality. Several studies demonstrated that RDW was independently associated with numerous adverse outcomes [10, 21, 22], and the results of our study also indicated that RDW was an independent predictor of mortality in critically ill patients with AKI.

AKI is a common, dangerous complication with a high mortality rate [23]. It is associated with local and systemic inflammation [24]. These inflammatory mediators are white blood cells, macrophages, components of endothelial cells, mast cells, fibroblasts, neutrophil, lymphocyte, and platelets [16, 25]. Diverse biomarkers have been determined to predict the prognosis of AKI in several studies [26, 27]. Similarly, our study showed that RDW was one of these biomarkers. RDW is an easily available biomarker and has been regarded as a useful predictive index for numerous diseases and organ dysfunctions [28–30]. Researchers in many observational studies [31, 32] have described an association between increased RDW and changes in inflammatory biomarkers. Therefore, the systemic inflammatory response probably helps to explain the potential link between RDW and the mortality in critically ill patients with AKI; however, the precise mechanisms for these relationships remain unknown.

In some studies, several possible explanations for these mechanisms have been proposed. Iron metabolism and bone marrow function were inhibited by inflammation [33]. Maturation and proliferation of erythrocyte were also inhibited by proinflammatory cytokines [34]. Furthermore, extent of inflammation played a negative impact on patient survival [35]. Moreover, the occurrence and progression of AKI were closely related to the inflammatory response. These revealed a close connection between RDW and AKI. In addition, oxidative stress resulted in a change in the size of red blood cells, reflected in an increase in RDW. Several studies demonstrated that oxidative stress, RDW, and all-cause mortality were closely related to each other [36, 37].

In subgroup analysis, we found the prognostic impact of RDW seemed to be weak in patients with baseline cardiac and cerebrovascular disease. On the other hand, the impact of RDW was significant in patients with respiratory failure, not in those with ARDS. In our study, subgroup analysis was based on the analysis of patients with AKI. If RDW independently predicted the prognosis of these diseases, the confounding factors that needed to be adjusted were different. Furthermore, the prognostic impact of RDW was not significant in patients requiring RRT. This was inconsistent with previous research [14]. To our knowledge, different sample sizes, research centers, and patient characteristics could lead to inconsistent results. Therefore, further studies, especially large prospective studies, are needed to confirm the relationship between RDW and adverse clinical outcomes.

The major strength of our study was that it was, to the best of our knowledge, the first study to comprehensively examine the relationship between RDW and mortality of AKI in critically ill patients. The RDW appears to be an independent prognostic marker in critically ill patients with AKI. Furthermore, the number of patients enrolled was very large.

This study also has some limitations. First, it had a single-center retrospective design, possibly affected by selection bias. Second, we measured RDW in patients only upon admission to the ICU and did not assess changes during the ICU stay. Third, insufficient information of the causative disease may lead to bias in multivariate analysis. Fourth, we did not know the patient's serum iron levels or whether erythropoietin use may have affected RDW values. Finally, the follow-up length of mortality varied. We only use hospital mortality and 30-day all-cause mortality, and this may affect the assessment of prognosis.

## 5. Conclusions

We found that RDW appeared to be an independent prognostic marker in critically ill patients with AKI and higher RDW was associated with increased risk of mortality in these patients. However, our findings need to be confirmed by large prospective studies with longer follow-up.

## Figures and Tables

**Table 1:** Characteristics of the study patients according to RDW.

Characteristics	RDW (%)			P value
	11.1-13.7	13.8-15.1	15.2-21.2	
Clinical parameters, n	4672	4664	4742	
Age, years	61.8 ± 18.8	67.7 ± 16.1	68.1 ± 15.2	<0.001
Female, n (%)	1736 (37.2)	2028 (43.5)	2228 (47.0)	<0.001
Ethnicity, n (%)				<0.001
White	3277 (70.1)	3321 (71.2)	3366 (71.0)	
Black	281 (6.0)	343 (7.3)	442 (9.3)	
Other	1114 (23.8)	1000 (21.4)	934 (19.7)	
RDW, %	13.1 ± 0.5	14.4 ± 0.4	17.3 ± 1.9	<0.001
BMI, kg/m^2^	28.1 ± 5.9	29.0 ± 6.8	28.9 ± 7.3	<0.001
SBP, mmHg	119.2 ± 16.1	118.3 ± 16.8	116.7 ± 16.9	<0.001
DBP, mmHg	61.2 ± 10.2	60.0 ± 10.6	58.4 ± 10.6	<0.001
Respiratory rate, beats/minute	18.7 ± 4.1	19.3 ± 4.3	19.5 ± 4.3	<0.001
Temperature, °C	37.0 ± 0.7	36.9 ± 0.7	36.8 ± 0.7	<0.001
Comorbidities, n (%)				
Congestive heart failure	498 (10.7)	815 (17.5)	1122 (23.67)	<0.001
Coronary artery disease	1466 (31.4)	1512 (32.4)	1244 (26.2)	<0.001
Atrial fibrillation	1200 (25.7)	1640 (35.2)	1779 (37.5)	<0.001
Chronic kidney disease	243 (5.2)	602 (12.9)	1007 (21.24%)	<0.001
Chronic liver disease	91 (2.0)	188 (4.0)	625 (13.2)	<0.001
COPD	86 (1.8)	136 (2.9)	135 (2.9)	0.001
Stroke	656 (14.0)	526 (11.3)	354 (7.5)	<0.001
Malignancy	417 (8.9)	686 (14.7)	1181 (24.9)	<0.001
Respiratory disease (non-COPD)	1672 (35.8)	1921 (41.2)	2187 (46.1)	<0.001
ARDS	89 (1.9)	103 (2.2)	108 (2.3)	0.413
Laboratory tests (missing data, %)				
WBC (0.04), 10^ 9^ /L	15.2 ± 6.5	14.8 ± 7.6	15.3 ± 13.3	<0.001
BUN (0.06), mg/dl,	24.2 ± 17.6	31.2 ± 22.9	40.5 ± 28.6	<0.001
Chloride (0.06), mmol/L	108.22 ± 6.27	108.18 ± 6.34	107.29 ± 6.96	<0.001
Bilrubin (48.46), mg/dl	1.1 ± 1.4	1.4 ± 2.7	3.1 ± 6.2	<0.001
Hematocrit (0.01), %	38.6 ± 5.4	36.4 ± 5.6	33.9 ± 5.3	<0.001
Hemoglobin (0.02), g/dl	13.0 ± 1.9	12.2 ± 1.9	11.1 ± 1.8	<0.001
Scoring systems				
SOFA	4.3 ± 2.9	5.2 ± 3.2	6.1 ± 3.4	<0.001
APSIII	42.8 ± 18.4	48.2 ± 19.7	55.3 ± 20.7	<0.001
Renal function				
Renal replacement therapy	155 (3.3)	318 (6.8)	667 (14.1)	<0.001
KDIGO stage, n (%)				<0.001
Stage 1	859 (18.39)	742 (15.91)	794 (16.74)	
Stage 2	993 (21.25)	899 (19.28)	815 (17.19)	
Stage 3	2820 (60.36)	3023 (64.82)	3133 (66.07)	

BMI: body mass index; SBP: systolic blood pressure; DBP: diastolic blood pressure; COPD: chronic obstructive pulmonary disease; ARDS: acute respiratory distress syndrome; WBC: white blood cell; BUN: blood urea nitrogen; SOFA: sequential organ failure assessment; APSIII: acute physiology score III; KDIGO Kidney Disease: Improving Global Outcomes.

**Table 2:** ORs (95% CIs) for mortality across groups of RDW.

RDW, %	Non-adjusted		Model I		Model II	
	OR (95%CIs)	P value	OR (95%CIs)	P value	OR (95%CIs)	P value
Hospital mortality						
Per 1 sd	1.24 (1.21, 1.27)	<0.0001	1.23 (1.20, 1.26)	<0.0001	1.12 (1.07, 1.17)	<0.0001
Quartiles						
11.1 - 13.4	1.0(ref)		1.0(ref)		1.0(ref)	
13.5 - 14.3	1.33 (1.14, 1.55)	0.0003	1.22 (1.05, 1.43)	0.0100	1.06 (0.82, 1.36)	0.6517
14.4 - 15.6	1.76 (1.52, 2.03)	<0.0001	1.56 (1.35, 1.81)	<0.0001	1.13 (0.89, 1.45)	0.3189
15.7 - 21.2	2.91 (2.53, 3.34)	<0.0001	2.66 (2.31, 3.06)	<0.0001	1.57 (1.22, 2.01)	0.0004
P trend	<0.0001		<0.0001		<0.0001	
30-day all-cause mortality						
Per 1 sd	1.24 (1.22, 1.27)	<0.0001	1.24 (1.21, 1.26)	<0.0001	1.13 (1.09, 1.18)	<0.0001
Quartiles						
11.1 - 13.4	1.0(ref)		1.0(ref)		1.0(ref)	
13.5 - 14.3	1.32 (1.14, 1.53)	0.0002	1.18 (1.02, 1.37)	0.0255	1.04 (0.82, 1.33)	0.7386
14.4 - 15.6	1.70 (1.47, 1.95)	<0.0001	1.44 (1.25, 1.66)	<0.0001	1.16 (0.91, 1.47)	0.2287
15.7 - 21.2	2.97 (2.60, 3.39)	<0.0001	2.65 (2.32, 3.02)	<0.0001	1.67 (1.31, 2.12)	<0.0001
P trend	<0.0001		<0.0001		<0.0001	

OR: odds ratio; CI: confidence interval.

Adjust I model; adjust for: age and gender.

Adjust II model; adjust for: age; gender; albumin; hemoglobin; liver disease; renal disease; malignancy; bilirubin; WBC; BUN; APSIII; SOFA; SIRS; RRT; temperature; Elixhauser.

**Table 3:** Subgroup analysis of the associations between RDW and hospital mortality.

	No. of patients	OR (95%CI)	P value
Age, year			
18-68.2	7014	1.07 (1.02, 1.13)	0.0080
68.3-91.4	7064	1.07 (1.02, 1.13)	0.0055
Gender			
F	5992	1.08 (1.02, 1.14)	0.0043
M	8086	1.05 (1.00, 1.10)	0.0657
CHF			
No	11643	1.08 (1.04, 1.12)	0.0002
Yes	2435	1.04 (0.95, 1.14)	0.3920
AKI stage			
Stage 1	2395	1.12 (1.01, 1.24)	0.0268
Stage 2	2707	1.09 (1.00, 1.18)	0.0444
Stage 3	8976	1.06 (1.01, 1.10)	0.0119
AF			
No	9459	1.08 (1.03, 1.12)	0.0011
Yes	4619	1.06 (0.99, 1.12)	0.0830
COPD			
No	13721	1.07 (1.03, 1.11)	0.0002
Yes	357	1.01 (0.77, 1.32)	0.9696
CAD			
No	9856	1.07 (1.03, 1.11)	0.0004
Yes	4222	1.03 (0.94, 1.12)	0.5476
Stroke			
No	12542	1.09 (1.05, 1.13)	<0.0001
Yes	1536	1.04 (0.92, 1.17)	0.4969
Respiratory failure			
No	8298	1.02 (0.96, 1.09)	0.4467
Yes	5780	1.09 (1.04, 1.14)	0.0002
Pneumonia			
No	9966	1.06 (1.01, 1.11)	0.0170
Yes	4112	1.08 (1.02, 1.14)	0.0076
ARDS			
No	13778	1.07 (1.03, 1.11)	0.0003
Yes	300	1.00 (0.80, 1.26)	0.9832
Liver			
No	13174	1.08 (1.04, 1.12)	0.0001
Yes	904	1.01 (0.93, 1.11)	0.7745
Vasoactive drug			
No	6993	1.04 (0.98, 1.10)	0.2382
Yes	7085	1.09 (1.04, 1.14)	0.0001
RRT			
No	12938	1.09 (1.05, 1.13)	<0.0001
Yes	1140	0.97 (0.89, 1.06)	0.4664
SIRS			
0	104	1.28 (0.81, 2.00)	0.2891
1	887	1.27 (1.13, 1.42)	<0.0001
2	2952	1.27 (1.20, 1.35)	<0.0001
3	5486	1.26 (1.21, 1.31)	<0.0001
4	4873	1.20 (1.15, 1.24)	<0.0001

CHF: congestive heart failure; AKI: acute kidney injury; AF: atrial fibrillation; COPD: chronic obstructive pulmonary disease; CAD: coronary artery disease; ARDS: acute respiratory distress syndrome; RRT: renal replacement therapy; SIRS: systemic inflammatory response syndrome.

## Data Availability

The clinical data used to support the findings of this study were supplied by Monitoring in Intensive Care Database III version 1.3 (MIMIC-III v.1.3). Although the database is publicly and freely available, researchers must complete the National Institutes of Health's web-based course known as Protecting Human Research Participants to apply for permission to access the database.

## Supplementary Materials

Supplementary Materials Supplementary file 1: the major SQL script used to extract data from MIMIC III.
