# Supplementary material for: The Association between Red Blood Cell Distribution Width and Mortality in Critically Ill Patients with Acute Kidney Injury
Source: Biomed Res Int. 2018 Sep 24;2018:9658216. doi: 10.1155/2018/9658216 (PMC6174796; doi:10.1155/2018/9658216)
Supplement: Supplementary Materials — Supplementary file 1: the major SQL script used to extract data from MIMIC III. [file 9658216.f1.docx]

The major SQL script used to extract data from mimicIII

SELECT

"public".rdw.rdw_start,

"public".icustay_detail11.gender,

"public".icustay_detail11.mortality,

"public".icustay_detail11.age,

"public".icustay_detail11.ethnicity,

"public".icustay_detail11.hospital_expire_flag,

"public".icustay_detail11.th_mortality_flag,

"public".icustay_detail11.ni_mortality_flag,

"public".icustay_detail11.t_hundred_mortality_flag,

"public".labsfirstday.aniongap_min,

"public".labsfirstday.aniongap_max,

"public".labsfirstday.albumin_min,

"public".labsfirstday.albumin_max,

"public".labsfirstday.bands_min,

"public".labsfirstday.bands_max,

"public".labsfirstday.bicarbonate_min,

"public".labsfirstday.bicarbonate_max,

"public".labsfirstday.bilirubin_min,

"public".labsfirstday.bilirubin_max,

"public".labsfirstday.creatinine_min,

"public".labsfirstday.creatinine_max,

"public".labsfirstday.chloride_min,

"public".labsfirstday.chloride_max,

"public".labsfirstday.glucose_min,

"public".labsfirstday.glucose_max,

"public".labsfirstday.hematocrit_min,

"public".labsfirstday.hematocrit_max,

"public".labsfirstday.hemoglobin_min,

"public".labsfirstday.hemoglobin_max,

"public".labsfirstday.lactate_min,

"public".labsfirstday.lactate_max,

"public".labsfirstday.platelet_min,

"public".labsfirstday.platelet_max,

"public".labsfirstday.potassium_min,

"public".labsfirstday.potassium_max,

"public".labsfirstday.ptt_min,

"public".labsfirstday.ptt_max,

"public".labsfirstday.inr_min,

"public".labsfirstday.inr_max,

"public".labsfirstday.pt_min,

"public".labsfirstday.pt_max,

"public".labsfirstday.sodium_min,

"public".labsfirstday.sodium_max,

"public".labsfirstday.bun_min,

"public".labsfirstday.bun_max,

"public".labsfirstday.wbc_min,

"public".labsfirstday.wbc_max,

"public".kdigo_stages_7day.aki_stage_7day,

"public".apsiii.apsiii,

"public".sofa.sofa,

"public".icustay_detail11.icustay_id,

"public".sirs.sirs,

"public".sapsii.sapsii,

"public".rrt.rrt,

"public".vitalsfirstday.heartrate_mean,

"public".vitalsfirstday.sysbp_mean,

"public".vitalsfirstday.diasbp_mean,

"public".vitalsfirstday.meanbp_mean,

"public".vitalsfirstday.resprate_mean,

"public".vitalsfirstday.tempc_mean,

"public".vitalsfirstday.spo2_mean,

"public".vitalsfirstday.glucose_mean,

"public".vaso_flag.vaso_flag,

"public".elixhauser_ahrq_score.elixhauser_sid30,

"public".icd.chf,

"public".icd.afib,

"public".icd.renal,

"public".icd.liver,

"public".icd.copd,

"public".icd.cad,

"public".icd.stroke,

"public".icd.malignancy,

"public".icd.respfail,

"public".icd.ards,

"public".icd.pneumonia

FROM

"public".rdw ,

"public".icustay_detail11 ,

"public".labsfirstday ,

"public".kdigo_stages_7day ,

"public".apsiii ,

"public".sofa ,

"public".sirs ,

"public".sapsii ,

"public".rrt ,

"public".vitalsfirstday ,

"public".vaso_flag ,

"public".elixhauser_ahrq_score ,

"public".icd

WHERE

"public".rdw.subject_id = "public".icustay_detail11.subject_id AND

"public".rdw.hadm_id = "public".icustay_detail11.hadm_id AND

"public".icustay_detail11.first_icu_stay = 'Y' AND

"public".icustay_detail11.age >= 18 AND

"public".icustay_detail11.icustay_id = "public".labsfirstday.icustay_id AND

"public".icustay_detail11.icustay_id = "public".kdigo_stages_7day.icustay_id AND

"public".kdigo_stages_7day.aki_7day = 1 AND

"public".icustay_detail11.icustay_id = "public".apsiii.icustay_id AND

"public".icustay_detail11.icustay_id = "public".sofa.icustay_id AND

"public".icustay_detail11.icustay_id = "public".sirs.icustay_id AND

"public".icustay_detail11.icustay_id = "public".sapsii.icustay_id AND

"public".icustay_detail11.icustay_id = "public".rrt.icustay_id AND

"public".icustay_detail11.icustay_id = "public".vitalsfirstday.icustay_id AND

"public".icustay_detail11.icustay_id = "public".vaso_flag.icustay_id AND

"public".icustay_detail11.los_icu >= 2 AND

"public".rdw.hadm_id = "public".elixhauser_ahrq_score.hadm_id AND

"public".rdw.hadm_id = "public".icd.hadm_id
